# Efficacy of a Self-Regulation–Based Electronic and Mobile Health Intervention Targeting an Active Lifestyle in Adults Having Type 2 Diabetes and in Adults Aged 50 Years or Older: Two Randomized Controlled Trials

**DOI:** 10.2196/13363

**Published:** 2019-08-02

**Authors:** Louise Poppe, Ilse De Bourdeaudhuij, Maïté Verloigne, Samyah Shadid, Jelle Van Cauwenberg, Sofie Compernolle, Geert Crombez

**Affiliations:** 1 Department of Movement and Sports Sciences Ghent University Ghent Belgium; 2 Department of Experimental Clinical and Health Psychology Ghent University Ghent Belgium; 3 Department of Endocrinology Ghent University Hospital Ghent Belgium; 4 Department of Public Health and Primary Care Ghent University Ghent Belgium

**Keywords:** eHealth, mHealth, physical activity, type 2 diabetes, self-regulation

## Abstract

**Background:**

Adopting an active lifestyle plays a key role in the prevention and management of chronic diseases such as type 2 diabetes mellitus (T2DM). Web-based interventions are able to alter health behaviors and show stronger effects when they are informed by a behavior change theory. *MyPlan 2.0* is a fully automated electronic health (eHealth) and mobile health (mHealth) intervention targeting physical activity (PA) and sedentary behavior (SB) based on the Health Action Process Approach (HAPA).

**Objective:**

This study aimed to test the short-term effect of *MyPlan 2.0* in altering levels of PA and SB and in changing personal determinants of behavior in adults with T2DM and in adults aged ≥50 years.

**Methods:**

The study comprised two randomized controlled trials (RCTs) with an identical design. RCT 1 was conducted with adults with T2DM. RCT 2 was performed in adults aged ≥50 years. Data were collected via face-to-face assessments. The participants decided either to increase their level of PA or to decrease their level of SB. The participants were randomly allocated with a 2:1 ratio to the intervention group or the waiting-list control group. They were not blinded for their group allocation. The participants in the intervention group were instructed to go through *MyPlan 2.0*, comprising 5 sessions with an interval of 1 week between each session. The primary outcomes were objectively measured and self-reported PA (ie, light PA, moderate-to-vigorous PA, total PA, number of steps, and domain-specific [eg, transport-related] PA) and SB (ie, sitting time, number of breaks from sitting time, and length of sitting bouts). Secondary outcomes were self-reported behavioral determinants for PA and SB (eg, self-efficacy). Separate linear mixed models were performed to analyze the effects of *MyPlan 2.0* in the two samples.

**Results:**

In RCT 1 (n=54), the PA intervention group showed, in contrast to the control group, a decrease in self-reported time spent sitting (*P*=.09) and an increase in accelerometer-measured moderate (*P*=.05) and moderate-to-vigorous PA (*P*=.049). The SB intervention group displayed an increase in accelerometer-assessed breaks from sedentary time in comparison with the control group (*P*=.005). A total of 14 participants of RCT 1 dropped out. In RCT 2 (n=63), the PA intervention group showed an increase for self-reported total PA in comparison with the control group (*P*=.003). Furthermore, in contrast to the control group, the SB intervention group decreased their self-reported time spent sitting (*P*=.08) and increased their accelerometer-assessed moderate (*P*=.06) and moderate-to-vigorous PA (*P*=.07). A total of 8 participants of RCT 2 dropped out.

**Conclusions:**

For both the samples, the HAPA-based eHealth and mHealth intervention, *MyPlan 2.0*, was able to improve only some of the primary outcomes.

**Trial Registration:**

ClinicalTrials.gov NCT03291171; http://clinicaltrials.gov/ct2/show/NCT03291171. ClinicalTrials.gov NCT03799146; http://clinicaltrials.gov/ct2/show/NCT03799146.

**International Registered Report Identifier (IRRID):**

RR2-10.2196/12413

## Introduction

The prevalence of chronic diseases, such as type 2 diabetes mellitus (T2DM), cardiovascular disease, and cancer, is high and rising [1,2]. Adopting an active lifestyle (ie, increasing physical activity [PA] and reducing sedentary behavior [SB]) plays an important role in the prevention and management of these diseases [3,4]. Indeed, adults are recommended to accumulate 150 min of moderate-to-vigorous PA (MVPA) [5] and minimize periods of prolonged sedentary time [6]. However, the majority of adults do not meet the guidelines considering PA and accumulate high levels of sitting time [4]. Even people for whom adopting an active lifestyle is considered a cornerstone in the management of their disease, such as people with T2DM, show high levels of physical inactivity and sedentary time [7,8]. Consequently, interventions targeting increases in PA and decreases in SB in adults with T2DM as well as in adults from the general population are needed.

As the number of internet and mobile phone users increases, interest in electronic health (eHealth) and mobile health (mHealth) interventions is growing [9]. eHealth and mHealth interventions offer several advantages as they can deliver fast and tailored information to large groups of individuals in a cost-effective way. Research examining the common effect of eHealth or mHealth interventions targeting PA or SB reports trivial-to-small and short-term effects [10-12]. Important points for improvement are better reporting of the theoretical basis as well as active ingredients (ie, the implemented behavior change techniques) of the intervention [10,12,13] and adopting objective measures to assess PA and SB [12].

Theory-based interventions delivered via the internet show stronger effects than internet-based interventions making less extensive or no use of theory (median d_+_=0.19) [14]. Self-regulation frameworks highlight the importance of bridging the intention-behavior gap by considering pre- as well as postintentional determinants of behavior change [15]. A review of Rhodes et al (2015) provides an overview of models incorporating pre- as well as postintentional determinants of PA [16]. The identified models showed considerable overlap in the proposed processes to bridge the intention-behavior gap. The results further showed that the health action process approach (HAPA) [17] was the most often used and independently tested framework. Indeed, the HAPA has been applied to alter the levels of PA and SB in clinical (including adults with T2DM [18]) and in nonclinical populations [19,20]. In recent years, this theoretical framework has also been used for developing Web-based behavioral interventions [21-24]. For example, *SmartMobiel*, an eHealth intervention informed by the HAPA-model was found to be effective in increasing PA in adults [25]. According to the HAPA, *risk perception*, *outcome expectancies*, *self-efficacy*, *intention*, *action planning*, *coping planning*, and *monitoring* are personal determinants playing a key role in behavior change.

*MyPlan 2.0* is a stand-alone HAPA-based eHealth and mHealth intervention comprising (1) a website offering weekly sessions to create and evaluate personal goals and (2) an optional mobile app providing daily support [26]. The program offers a module targeting increases in PA and a module targeting reductions in SB. The users autonomously select which behavior they will focus on. This was done because of two reasons. First, PA and SB are considered distinct rather than opposite behaviors, each having a unique contribution to people’s mental and physical health [27]. Indeed, one might reach the health norms regarding PA and still show high levels of SB and vice versa. Second, the self-regulation framework emphasizes the importance of goal ownership and highlights the need to let people select goals that they can relate with [15]. The program aims to alter behavior by targeting the HAPA-based personal determinants of behavior. As obtaining large changes in behavior might take longer than the length of the program, it is important to also assess whether the intervention altered the targeted personal determinants for behavior. These personal determinants could be altered on a shorter term and, according to the HAPA [28], changes in the personal determinants will result in changes in behavior.

The aim of this study was to test the efficacy of *MyPlan 2.0* to alter behavior (primary outcome) and behavioral determinants (secondary outcome) in adults with T2DM. The research protocol for the randomized controlled trial (RCT) in patients with T2DM was published [26]. However, we encountered difficulties in recruiting participants with T2DM. For that reason, it was decided to recruit an additional group of participants from the general population from a similar age cohort as the population with T2DM. Consequently, the participants of RCT 1 were adults diagnosed with T2DM and the participants of RCT 2 were adults aged 50 years or older.

## Methods

### Hypotheses

Similar hypotheses were formulated for both RCTs. Regarding PA, we hypothesized that *MyPlan 2.0* would have a positive effect on self-reported and objectively measured levels of total PA, MVPA, and light PA (LPA) in the PA intervention group compared with the control group. Regarding sedentary behavior, we hypothesized that *MyPlan 2.0* would reduce self-reported and objectively measured total sitting time in the SB intervention group compared with the control group. Furthermore, as the intervention focused on limiting sedentary time as well as interrupting periods of prolonged sitting, we expected to find an increase in breaks from sedentary time and a decrease in the length of the sedentary bouts in the intervention group targeting SB compared with the control group. Regarding the personal determinants, we expected that *MyPlan 2.0* would increase the participants’ self-efficacy, outcome expectations, intention, action planning, coping planning, and self-monitoring. No hypotheses regarding the participants’ risk perception were made as *MyPlan 2.0* did not specifically target this personal determinant [26].

### Study Design and Procedure

In total, 2 RCTs with a parallel group design were conducted to investigate the effect of *MyPlan 2.0* on PA, SB, and HAPA-based determinants. The protocol was preregistered [26]. The a priori power analysis suggested a sample size of 96 participants. Adults with T2DM were recruited via the Ghent University Hospital and the Damian General Hospital (Ostend). However, recruitment via the hospitals was slow. Therefore, in contrast with the recruitment process described in the protocol, we also advertised the study via the Flemish Diabetes Association and in adults with T2DM who participated in the previous research of the involved research groups. The sample of adults aged ≥50 years was recruited via advertisements in local newspapers and via snowball sampling. For both samples, the inclusion criteria were (1) being literate in the Dutch language to engage in the intervention, (2) being computer literate, (3) having internet access, and (4) not having participated in the qualitative study about *MyPlan 2.0*. Additional inclusion criteria to participate in RCT 1 were being diagnosed with T2DM since at least 1 month and being 18 years or older, whereas the participants of RCT 2 were required to be aged 50 years or older.

**Figure 1:**
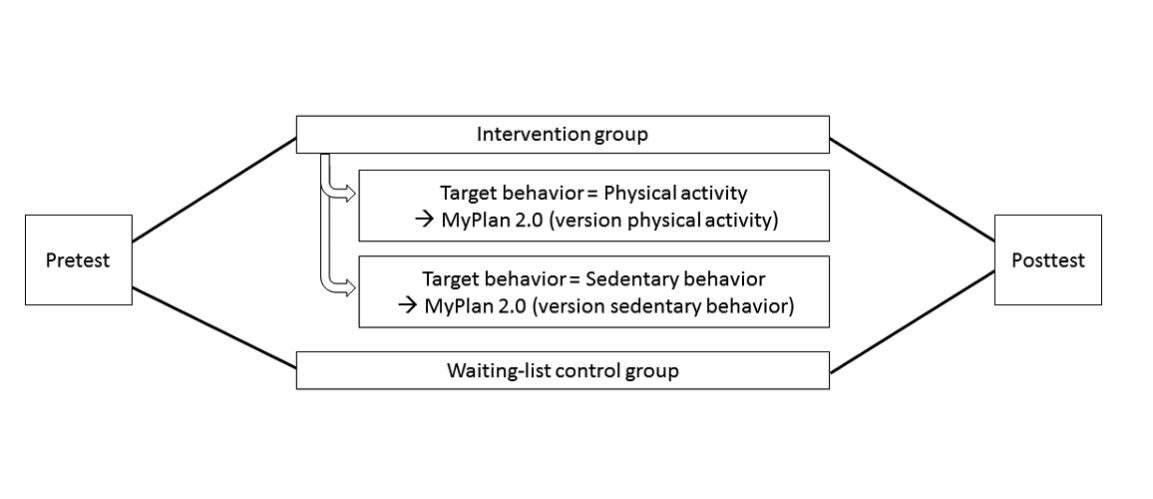
Design of the randomized controlled trials.

Figure 1 displays the design of the RCTs. After enrollment, the participants were visited by one of the researchers. During the home visit, the researcher explained the difference between PA and SB and asked the participants to select a target behavior (ie, increasing PA or decreasing SB). The participants completed questionnaires and their weight and waist circumference were assessed. The participants were instructed to wear an accelerometer for 10 consecutive days starting the day after the home visit. After these 10 days, the participants were allocated by LP to the intervention or the waiting-list control group using a 2:1 ratio. This was done via a random number generator. The participants allocated to the waiting-list control group were informed about their allocation and instructed to continue with their life as usual. The participants allocated to the intervention group received access to the MyPlan 2.0 website and the mobile app. The participants who selected to focus on their level of PA were guided to the version targeting PA (PA intervention group), whereas the participants who selected to alter their level of SB were guided to the version targeting SB (SB intervention group). They were instructed to go through each of the weekly sessions (5 in total) offered by the website. The involved researchers inspected the logfile of the website to check whether the participants logged in for each session. The participants who forgot to log in were contacted by a researcher via email and informed about the next session. If the participant did not respond, he or she was contacted via telephone. As having a smartphone was not an inclusion criterion, it was not obligatory to use the mobile app. To monitor any adverse effects (eg, hypoglycemia), all participants were weekly phoned by a member of the research team. No coaching took place during these phone calls.

After completing all sessions (PA and SB intervention groups) or the 5-week waiting period (control group), a second home visit was arranged. During this second home visit, the participants completed the same assessments as at baseline. The participants who decided to leave the study were contacted by one of the researchers and asked if they were willing to complete a questionnaire assessing potential reasons for attrition. Except during the pretest (the participants were allocated to a group after the pretest), neither the participants nor researchers assessing the outcome variables were blinded.

All data were collected between January and September 2018. No changes regarding bug fixes, downtimes, or content changes to the Web-based program occurred after trial commencement. The RCTs were approved by the Committee of Medical Ethics of the Ghent University Hospital (Belgian registration numbers: B670201732566 for RCT 1 and BE670201731996 for RCT 2).

#### MyPlan 2.0

*MyPlan 2.0* is a free fully automated HAPA-based eHealth and mHealth intervention comprising a website and an optional mobile app. Its precursor, *MyPlan 1.0*, showed high levels of attrition. Several user-based studies were performed to better adapt *MyPlan 2.0* to the users’ needs [29-32]. Multimedia Appendix 1 provides an overview of the lessons learned from each of these studies and describes how these findings guided the adaptations to *MyPlan 2.0*. The program offers a number of behavior-change techniques aiming to influence the users’ HAPA-based personal determinants for change. The used techniques are mentioned below and labelled according to the taxonomy of behavior change techniques of Michie et al [33]. Multimedia Appendix 2 provides screenshots of the website and the mobile app.

##### The Website

**Figure 2:**
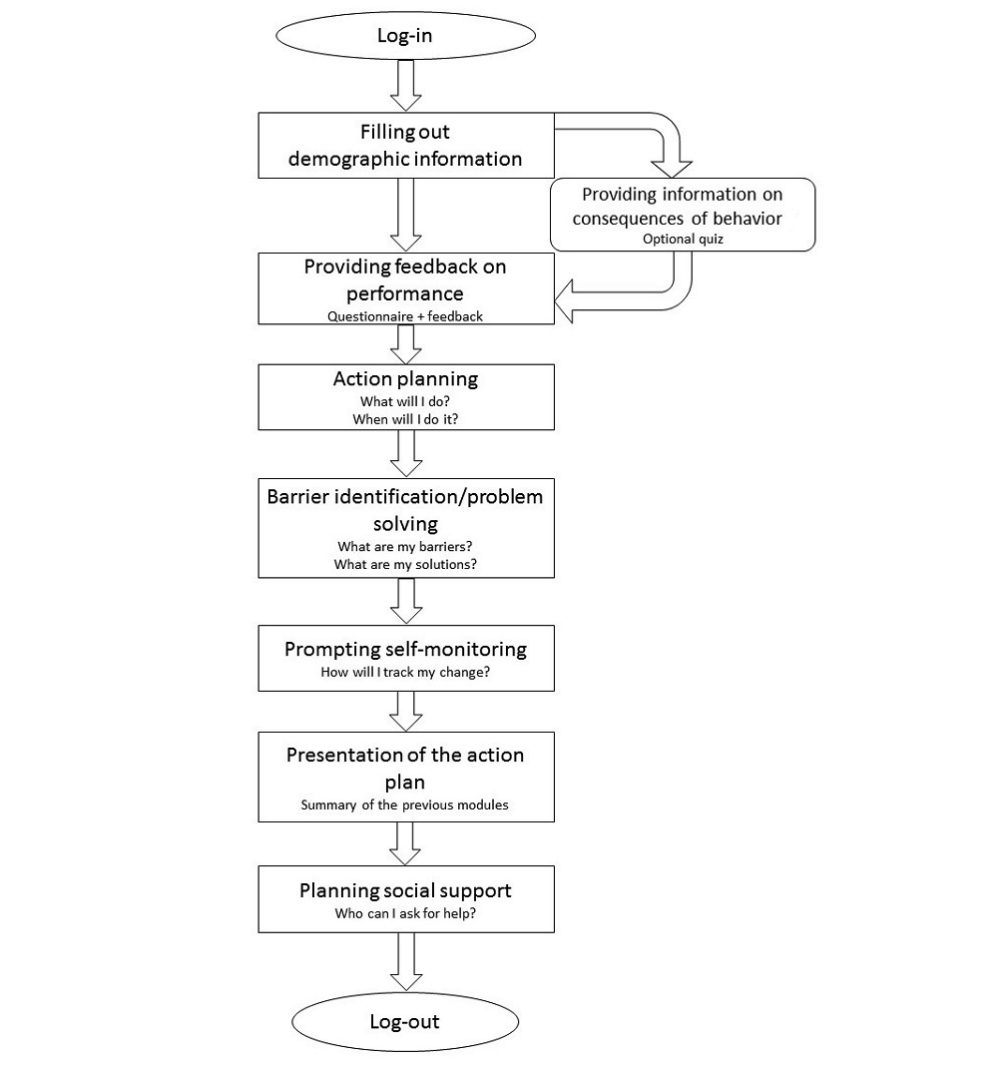
Flow of the first session.

The website part of *MyPlan 2.0* was created using LifeGuide [34] and offers 5 sessions with a period of 1 week between each session. The two versions of the program (one targeting increases in PA and one targeting reductions in SB) have an identical structure and offer the same self-regulation techniques. During the first session, the users create a profile, complete an optional quiz regarding the benefits of the chosen health behavior (ie, increasing PA or reducing SB, *providing information on consequences of behavior)*, fill out a questionnaire assessing their current level of PA or SB and receive tailored feedback (*providing feedback on performance*), create a personal action plan to alter the chosen health behavior *(action planning)*, foresee potential barriers and search for solutions *(barrier identification/problem solving)*, and select how they will monitor their behavior *(prompting self-monitoring of behavior).* At the end of the first session, the users’ answers are summarized in a printable action plan and they are offered optional information about how they can obtain support from their partner, friends, family, or colleagues *(exploring social support)*. Figure 2 shows the flow of the first session.

**Figure 3:**
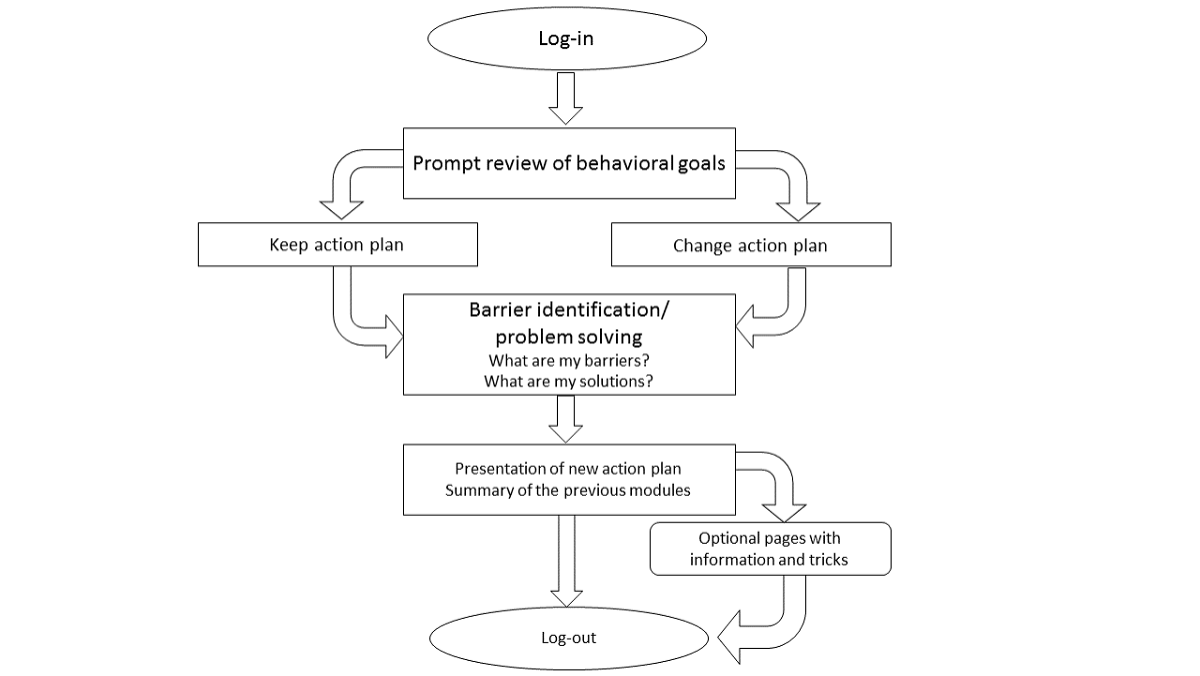
Flow of the follow-up sessions.

After 1 week, the users receive an email to start the second session. The follow-up sessions (ie, sessions 2-5) have a similar structure. After logging in, the users are asked to what extent they reached the goal set in the previous session *(prompting review of behavioral goals)* and whether they would like to keep or adapt this goal. When choosing the latter, the user is guided to the action planning section. All users again foresee potential barriers to reach the goal and search for solutions. Finally, their answers are summarized in a printable action plan and the users are optionally offered additional tips and tricks (eg, *try to take the stairs instead of using the elevator*) to become more physically active or less sedentary. Figure 3 depicts the flow of the follow-up sessions.

##### The Mobile App

The mobile app comprises 5 modules through which the users can freely navigate. The first module supports users in monitoring their behavior *(prompting self-monitoring of behavior)*. Every evening, the users receive a notification to report the extent to which they were able to be more physically active or to sit less (eg, *not at all*, *not*, *a little*, *well*, and *very well*). These entries are then shown in a graph displaying all responses of the week. The second module allows the users to review their weekly goals (created on the website) and make adaptations to these goals *(action planning)*. The option to review potential problems and their solutions is offered in the third module *(barrier identification/problem solving)*. In the fourth module, the users can perform quizzes on the benefits of being more physically active or less sedentary *(providing information on the consequences of behavior).* Finally, the users can collect points by visiting the website, completing quizzes, and monitoring their behavior. By collecting these points, they could earn the victory cups implemented in the mobile app. This gamification element was added to increase engagement with the mobile app.

### Measurements

#### Participant Characteristics

An ad hoc questionnaire assessed age, sex, height, civil status, level of education, and time since diagnosis (only for the participants with T2DM). The participants who completed college or university were considered highly educated.

The participants’ weight and waist circumference were assessed using a Seca weighting scale (type 813) and a Seca measuring tape. Waist circumference was measured at the lowest rib margin and the iliac crest at the midaxillary line. During each testing wave, the participants’ weight and waist circumference were measured twice. In case the difference between the two measurements was >100 grams or >1 cm, the measurement was performed a third time. The mean of the measurements was calculated as the final score.

#### Primary Outcomes

The long version of the international physical activity questionnaire (IPAQ) [35] (translated into Dutch) assesses self-reported PA of the past week in 4 domains (work, transport, household, and leisure time) and provides indicators for work-related PA, transport-related PA, household-related PA, leisure-related PA, total PA, vigorous-intensity PA (VPA), and MVPA per week. The IPAQ has good reliability (intraclass range 0.46-0.96) and a fair-to-moderate criterion validity (Spearman rho between .30 and .37) [35]. As the IPAQ overestimates PA [36], the data were truncated according to the method described by Dubuy et al [37]. The LASA (Longitudinal Aging Study Amsterdam) sedentary behavior questionnaire [38], which has moderate reliability (intraclass=0.71) and moderate validity (Spearman rho=.35), was used to assess usual total sedentary time on weekdays. Data were truncated at a maximum of 16 hours of sitting time a day [39]. Both questionnaires were conducted via an interview by the visiting researcher.

ActiGraph accelerometers (type GT3X+), shown to be reliable and valid [40-43], were used to assess the participants’ number of breaks from sedentary time, average length of the sedentary bouts, total sedentary time, number of steps, LPA, moderate PA (MPA), VPA, MVPA, and total PA. The participants were instructed to wear the accelerometer on the right hip during waking hours but to remove it for water-based activities (eg, showering). ActiLife 6.13.3 software (ActiGraph, Fort Walton Beach, FL, USA) was used to initialize the accelerometers and process the data. The epoch was set at 60 seconds and nonwear time was calculated as ≥60 min of consecutive 0 counts. The participants’ accelerometer data were included in the study when they had at least 4 valid days including 1 weekend day (with valid defined as ≥10 hours of wearing time) [44]. Using the cut points described by Freedson et al [45], each minute of wear time was categorized as sedentary (0-99 counts per min [CPM]), LPA (100-1951 CPM), MPA (1952-5724 CPM), VPA (5725-9498 CPM), or MVPA (≥1952 CPM). Total PA was calculated by combining LPA and MVPA. A bout of sedentary time was considered a period of at least 10 consecutive min <99 counts with zero tolerance allowed. A break from a sedentary bout was defined as a transition from <99 CPM to >99 CPM between 2 sedentary bouts.

#### Secondary Outcomes

The participants’ HAPA-based personal determinants for behavior change (ie, self-efficacy, risk perceptions, outcome expectations, intention, action planning, coping planning, and self-monitoring) were measured using multiple items with a minimum of 3 items per determinant. To select these items, a large number of items measuring HAPA determinants were presented to 11 experts in the self-regulation framework. All experts indicated for each item whether or not it measured the presented HAPA determinant and how certain they were of their answer [46]. On the basis of their responses, a discriminant content validity method was used [46] and the best scoring items were selected. To assure comprehensibility of these items, cognitive interviews were conducted with 4 adults (mean age 58.3, SD 6.5, 3 women, 2 having T2DM, and 2 with a college degree or higher). On the basis of the results of these interviews, the final items were selected and adapted. Each item was assessed using 10 answer options ranging from *completely disagree* to *completely agree*. For each personal determinant, a mean score (potential range 1-10) was calculated.

### Statistical Analysis

The data from both RCTs were analyzed separately using R version 3.2.5 [47]. Nevertheless, the analyses were similar for both the RCTs.

Group comparability at baseline between the two intervention groups (PA intervention group and SB intervention group) and the control group was investigated using a 1-way analysis of variance (for the quantitative variables) and chi-square tests (for the qualitative variables). *T* tests and chi square tests were used to perform the dropout analysis. Linear mixed models (2 levels: repeated measures clustered within the participants) were performed using the *lme4-package* [48] to investigate the intention-to-treat effect of *MyPlan 2.0* on levels of PA, SB, and the personal determinants [49]. In contrast to the multivariate analysis of variance, the linear mixed model can easily handle missing data in repeated measures [50]. Furthermore, mixed models without ad hoc imputation provide equal or more power than mixed models with ad hoc imputation [51]. In the protocol, we stated that we would consider the participants’ choice of target behavior (ie, PA or SB) as moderator. However, because we were not able to recruit large enough samples, we decided to perform the analyses on the behavioral outcomes with a group variable (ie, the PA intervention group, the SB intervention group, and the control group).

All participants filled out one version of the HAPA-based determinants (ie, the version focusing on PA or the version focusing on SB). As described in the protocol, we planned to account for this issue by considering the choice of target behavior (ie, PA or SB) as moderator. However, considering the small sample sizes, we decided to combine the PA intervention group and the SB intervention group as one intervention group for analyzing the effect on the personal determinants. By doing so, we considered these outcome variables as personal determinants regarding the chosen health behavior rather than personal determinants regarding increasing PA or decreasing SB.

Owing to the low prevalence of accelerometer-based VPA (no VPA at baseline was detected in 93% (50/54) of the sample in RCT 1 and in 63% (40/63) of the sample in RCT 2), self-reported VPA (no self-reported VPA at baseline was detected in 80% (43/54) of the sample in RCT 1 and in 75% (47/63) of the sample in RCT 2), and self-reported work-related PA (no self-reported work-related PA at baseline in 69% (37/54) of the sample in RCT 1 and in 67% (42/63) of the sample in RCT 2) in both samples, these specific outcome variables were not analyzed.

Distribution of the dependent variables was first checked using Shapiro-Wilk tests. Normally distributed dependent variables were analyzed using the *lmer* function of the *lme4-package* [48]. For non-normally distributed variables, we compared models with different variance and link functions (ie, Gaussian with identity, gamma with log, gamma with identity, Poisson with log, and negative binomial with log) using the Bayesian information criterion (BIC). For each dependent variable, we selected the model providing the lowest BIC value. By exploring the interaction between time and group (ie, intervention vs control), the effect of the intervention on the dependent variable was assessed. The beta values for *time×group* reported in the results section describe the difference between the change in the intervention group and the change in the control group. Consequently, these values represent the intervention effect for each dependent variable. *P* values <.05 were considered statistically significant, whereas *P* values between .05 and .10 were considered borderline significant.

Effect sizes were calculated for each of the dependent variables in both samples [52]. As recommended by Morris [53], the pooled pretest standard deviation was used to estimate the effect sizes.

## Results

The results of the two RCTs are reported separately. The first section will describe the results of the RCT with the sample with T2DM (RCT 1), whereas the second section will describe the results of the RCT with the sample aged ≥50 years (RCT 2).

### Randomized Controlled Trial 1

**Figure 4:**
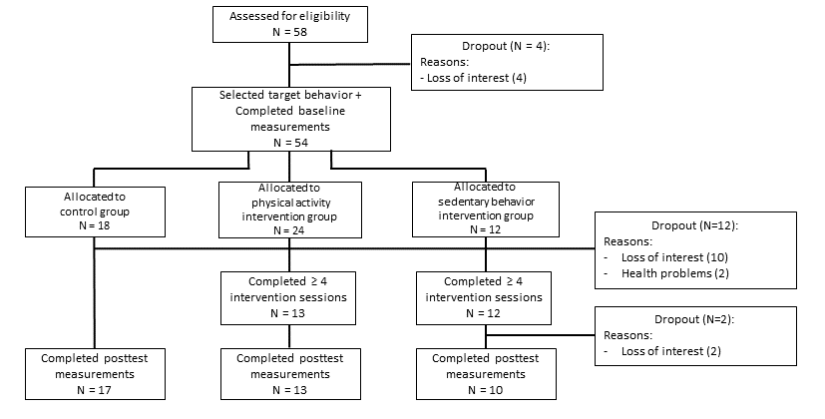
Flow of the sample of randomized controlled trial 1.

Figure 4 shows the flow of the participants with T2DM. A total of 58 participants agreed to participate in the study. Of this sample, 18 participants were recruited via the Ghent University Hospital, 8 via the Damian General Hospital, 24 via the Flemish Diabetes Association, and 8 via previous studies. As we do not know how many patients saw the advertisements, the response rate could not be calculated. Out of them, 4 participants dropped out before completing the baseline measurements. Consequently, the data of 54 participants were analyzed. Of the 14 participants who dropped out before completing 4 sessions, only 3 participants (all belonging to the control group) were willing to complete the questionnaire assessing specific reasons for attrition. Among them, 1 participant indicated that he doubted to participate at the beginning of the study and 2 participants indicated that drastic changes in their life occurred while participating. Finally, 1 participant indicated that the high number of research-related questionnaires frustrated her.

The participants’ baseline characteristics are provided in Table 1. At baseline, 32 participants decided to focus on PA (24 of these participants were later allocated to the intervention group) and 22 participants chose to focus on SB (12 of these participants were later allocated to the intervention group). Consequently, the PA intervention group comprised 24 participants and the SB intervention group comprised 12 participants. No significant baseline differences in sociodemographic characteristics were found among the PA intervention group, the SB intervention group, and the control group. Of the participants, 7 used the optional mobile app. The dropout analyses indicated that the participants allocated to the intervention group (χ^2^_1_=4.35, *P*=.04) were more likely to dropout. No significant differences between completers and dropouts were found for age, sex, level of education, body mass index (BMI), time since diagnosis, total PA at baseline (accelerometer-measured), or sedentary time at baseline (accelerometer-measured).

Table 2 displays the means and standard deviations for each of the behavioral outcomes in the three groups. Table 3 provides the time-by-group interactions and effect sizes for each of the behavioral outcomes. A borderline significant intervention effect favoring the PA intervention group was found for self-reported total daily sitting time (*P*=.09) and accelerometer-assessed MPA (*P*=.05) and MVPA (*P*=.049). A significant intervention effect favoring the SB intervention group was found for accelerometer-assessed daily breaks from sedentary time (*P*=.005). No intervention effects were found for self-reported total transport-related PA, self-reported total household-related PA, self-reported total leisure-related PA, self-reported total PA, self-reported MVPA, accelerometer-assessed length of the sedentary bouts, accelerometer-assessed sedentary time, accelerometer-assessed LPA, accelerometer-assessed total PA, or accelerometer-assessed daily steps.

Table 4 displays the time-by-group interactions and effect sizes for the personal determinants in RCT 1. Significant intervention effects favoring the control group were found for self-efficacy (*P*=.01) and risk perception (*P*=.03). A borderline significant intervention effect favoring the intervention group was found for action planning (*P*=.08). Finally, a significant time*group interaction effect favoring the intervention group was found for self-monitoring (*P*=.008). No intervention effects were found for outcome expectancies, coping planning, or intention.

**Table 1:** Baseline characteristics of the sample of randomized controlled trial 1.

Baseline characteristics	Total sample (N=54)	CG^a^ (n=18)	IG^b^–PA^c^ (n=24)	IG–SB^d^ (n=12)	*F* or χ^2^ (df)	*P* value
Age (years), mean (SD)	62.67 (8.40)	64.89 (8.62)	62.91 (7.16)	58.92 (9.52)	1.90^e^ (2, 49)	.16
Males, n (%)	34 (63)	9 (50)	17 (71)	8 (67)	2.01^f^ (2)	.37
University/college education, n (%)	29 (54)	12 (67)	10 (42)	7 (58)	1.85^f^ (2)	.40
Body mass index (kg/m²), mean (SD)	30.84 (5.66)	30.51 (6.86)	30.86 (5.35)	31.25 (4.73)	0.06^e^ (2, 48)	.94
Waist circumference (cm), mean (SD)	109.23 (14.08)	108.14 (18.38)	109.16 (11.09)	110.99 (13.06)	0.14^e^ (2,51)	.87
Time since diagnosis (months), mean (SD)	129.78 (83.09)	157.69 (67.08)	100.18 (87.25)	146.83 (82.94)	2.73^e^ (2,47)	.08

^a^CG: control group.

^b^IG: intervention group.

^c^PA: physical activity.

^d^SB: sedentary behavior.

^e^*F* value.

^f^χ^2^ value.

**Table 2:** Means and standard deviations for each of the behavioral outcomes in the three groups in randomized controlled trial 1.

Behavioral outcomes		CG^a^, mean (SD)		IG^b^–PA^c^, mean (SD)		IG–SB^d^, mean (SD)	
		Pre	Post	Pre	Post	Pre	Post
**LASA^e^ questionnaire**							
	Total sitting time (min/day)	553.06 (174.05)	567.94 (211.84)	592.83 (232.52)	470.38 (185.23)	599.17 (133.58)	579.44 (188.84)
**IPAQ^f^**							
	Total transport-related PA (min/day)	18.02 (24.61)	31.30 (36.60)	13.90 (25.70)	29.34 (24.61)	35.54 (35.30)	26.50 (23.16)
	Total household-related PA (min/day)	45.28 (71.89)	46.89 (62.34)	63.33 (71.60)	62.96 (56.18)	44.17 (78.15)	67.29 (92.89)
	Total leisure-related PA (min/day)	16.35 (18.76)	38.45 (59.88)	19.08 (25.59)	30.61 (41.58)	38.63 (46.27)	49.86 (74.39)
	Total PA (min/day)	86.07 (70.54)	124.50 (96.02)	113.81 (90.52)	143.83 (91.48)	137.98 (99.89)	150.07 (100.48)
	Moderate-to-vigorous physical activity (min/day)	17.86 (24.37)	61.98 (75.83)	53.19 (72.03)	48.67 (41.35)	47.26 (67.18)	63.57 (76.93)
**Accelerometer**							
	Number of breaks per day	16.63 (2.25)	15.65 (2.87)	15.51 (3.08)	14.64 (2.39)	16.88 (1.69)	17.50 (1.45)
	Length of sedentary bouts (min/day)	22.90 (2.70)	23.46 (2.01)	22.34 (2.44)	22.82 (2.64)	22.80 (1.57)	23.22 (1.14)
	Sedentary time (min/day)	544.52 (56.24)	537.77 (81.01)	528.09 (80.79)	498.09 (43.81)	551.39 (56.66)	555.21 (44.39)
	Light physical activity (min/day)	239.34 (73.14)	231.51 (76.84)	238.13 (67.23)	244.46 (71.05)	218.87 (54.49)	215.69 (37.91)
	Moderate physical activity (min/day)	23.14 (12.93)	19.36 (14.77)	17.02 (15.70)	25.50 (15.77)	20.15 (13.92)	19.13 (14.06)
	Moderate-to-vigorous physical activity (min/day)	23.20 (12.92)	19.36 (14.77)	17.07 (15.68)	25.50 (15.77)	20.23 (13.87)	19.38 (13.81)
	Total PA	262.54 (78.58)	267.17 (83.11)	255.19 (69.00)	269.95 (78.24)	239.10 (48.83)	235.07 (33.89)
	Daily steps	6203.10 (2284.41)	6292.05 (2480.44)	5364.39 (2219.28)	6549.71 (2313.67)	6083.88 (1343.30)	6001.03 (1107.26)

^a^CG: control group.

^b^IG: intervention group.

^c^PA: physical activity.

^d^SB: sedentary behavior.

^e^LASA: Longitudinal Aging Study Amsterdam.

^f^IPAQ: international physical activity questionnaire.

**Table 3:** Time-by-group interactions and effect sizes for each of the behavioral outcomes in randomized controlled trial 1.

Behavioral outcomes		Time×group PA^a^ (ref: pre×CG^b^), beta (SE)	ES^c^ (IG^d^−PA vs CG)	Time×group SB^e^ (ref: pre×CG), beta (SE)	ES (IG−SB vs CG)
**LASA^f^ questionnaire**					
	Total sitting time (min/day)^g^	−102.50 (59.32)^h^	−0.65	−4.61 (66.59)	−0.22
**IPAQ^i^**					
	Total transport-related PA (min/day)^j^	0.19 (0.92)	0.09	−0.85 (1.05)	−0.76
	Total household-related PA (min/day)^j^	−0.04 (0.96)	−0.03	0.39 (1.10)	0.29
	Total leisure-related PA (min/day)^j^	−0.38 (0.99)	−0.46	−0.60 (1.13)	−0.33
	Total PA (min/day)^j^	−0.14 (0.52)	0.23	−0.29 (0.60)	−0.31
	Moderate-to-vigorous physical activity (min/day)^j^	−1.33 (0.85)	−0.85	−0.95 (0.97)	−0.60
**Accelerometer**					
	Number of breaks per day^k^	0.28 (0.63)	0.04	1.93 (0.69)^l^	0.77
	Length of sedentary bouts (min/day)^k^	−0.50 (0.59)	−0.03	−0.46 (0.62)	−0.06
	Sedentary time (min/day)^k^	−19.71 (23.92)	−0.32	19.91 (25.00)	0.19
	Light physical activity (min/day)^g^	7.02 (14.70)	0.20	−5.07 (14.94)	0.07
	Moderate physical activity (min/day)^j^	0.37 (0.19)^h^	0.86	0.02 (0.92)	0.21
	Moderate-to-vigorous physical activity (min/day)^j^	0.37 (0.19)^m^	0.84	0.06 (0.19)	0.39
	Total PA^g^	11.09 (15.47)	0.002	−4.54 (15.73)	−0.13
	Daily steps^g^	499.46 (543.12)	0.49	−302.11 (555.83)	−0.09

^a^PA: physical activity.

^b^CG: control group.

^c^ES: effect size.

^d^IG: intervention group.

^e^SB: sedentary group.

^f^LASA: Longitudinal Aging Study Amsterdam.

^g^Gaussian (identity).

^h^*P*<.10.

^i^IPAQ: international physical activity questionnaire.

^j^Gamma (log).

^k^Gamma (identity).

^l^*P*<.01.

^m^*P*<.05.

**Table 4:** Time-by-group interactions and effect sizes for the personal determinants in randomized controlled trial 1.

Personal determinants	CG^a^, mean (SD)		IG^b^, mean (SD)		Time×group (ref: pre×CG), beta (SE)	ES^c^
	Pre	Post	Pre	Post		
Self-efficacy^d^	5.68 (1.98)	6.88 (1.22)	6.35 (1.81)	7.23 (1.61)	−1.24 (0.48)^e^	−0.17
Outcome expectancies^d^	7.03 (1.51)	8.01 (1.10)	7.62 (1.63)	8.04 (0.96)	−0.09 (0.42)	−0.35
Risk perception^f^	4.46 (1.81)	5.10 (1.71)	5.16 (2.19)	4.74 (92.05)	−1.17 (0.53)^e^	−0.51
Action planning^f^	5.41 (1.99)	5.29 (2.21)	5.19 (2.32)	6.09 (2.05)	1.18 (0.65)^g^	0.46
Coping planning^d^	3.52 (2.43)	4.69 (2.22)	3.95 (2.66)	5.77 (2.32)	0.79 (0.54)	0.25
Intention^f^	6.87 (2.83)	7.88 (1.26)	7.82 (2.11)	8.06 (1.81)	−0.87 (0.76)	−0.32
Monitoring^h^	4.65 (3.21)	4.84 (2.98)	3.46 (2.61)	5.17 (2.41)	0.47 (0.18)^i^	0.54

^a^CG: control group.

^b^IG: intervention group.

^c^ES: effect size.

^d^Gamma (identity).

^e^*P*<.05.

^f^Gaussian (identity).

^g^*P*<.10.

^h^Gamma (log).

^i^*P*<.01.

### Randomized Controlled Trial 2

**Figure 5:**
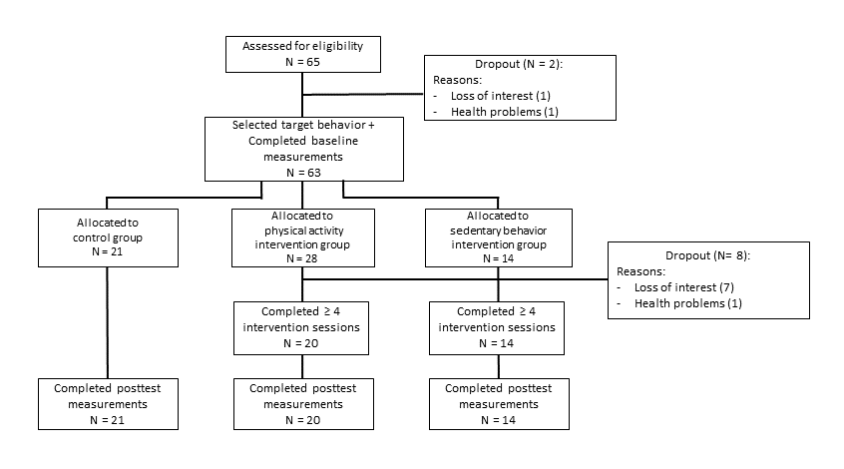
Flow of the sample of randomized controlled trial 2.

Figure 5 shows the flow of the participants. A total of 65 participants agreed to participate in the study. As we do not know how many people saw the advertisements, the response rate could not be calculated. Of them, 2 participants dropped out before completing the baseline measurements. Consequently, the data of 63 participants were analyzed. Of the 8 participants who dropped out before completing 4 sessions, only 1 was willing to complete the questionnaire assessing specific reasons for attrition. The participant indicated that *MyPlan 2.0* did not meet her expectations and that her friends or family did not respond positively to her participation in the study. Furthermore, she indicated that the high number of research-related questionnaires frustrated her.

The baseline characteristics of the participants are provided in Table 5. At baseline, 46 participants decided to focus on PA (28 of these participants were later allocated to the PA intervention group) and 17 participants chose to focus on SB (14 of these participants were later allocated to the SB intervention group). Consequently, the PA intervention group comprised 28 participants and the SB intervention group comprised 14 participants. No significant baseline differences in sociodemographic characteristics were found among the PA intervention group, the SB intervention group, and the control group. Of the participants, 5 used the optional mobile app. The dropout analyses indicated that the participants with a lower level of education (ie, no college or university degree) (χ^2^_1_=3.2; *P*=.07) and those allocated to the intervention group (χ^2^_1_=3.0; *P*=.08) were more likely to drop out. No significant dropout effects were found for age, sex, BMI, total PA at baseline (accelerometer-measured), or sedentary time at baseline (accelerometer-measured).

Table 6 displays the means and standard deviations for each of the behavioral outcomes in the three groups. Table 7 provides the time-by-group interactions and effect sizes for each of the behavioral outcomes. A significant intervention effect favoring the PA intervention group was identified for self-reported total PA (*P*=.003). Borderline significant intervention effects favoring the SB intervention group were found for self-reported daily sitting (*P*=.08), MPA (*P*=.06), and MVPA (*P*=.07). No intervention effects were detected for the outcome variables self-reported total transport-related PA, self-reported total household-related PA, self-reported total leisure-related PA, accelerometer-assessed MVPA, accelerometer-assessed number of breaks per day, accelerometer-assessed length of the sedentary bouts, accelerometer-assessed sedentary time, accelerometer-assessed LPA, accelerometer-assessed total PA, or accelerometer-assessed daily steps.

Table 8 displays the time-by-group interactions and effect sizes for the personal determinants in RCT 2. As described above, the PA intervention group and the SB intervention group were considered as one group to analyze the effect on the personal determinants. For coping planning, a significant intervention effect favoring the intervention group was found (*P*<.001). Furthermore, borderline significant intervention effects favoring the intervention group were found for intention (*P*=.07), self-efficacy (*P*=.05), and monitoring (*P*=.09). No intervention effect was found for outcome expectancies, risk perception, or action planning.

**Table 5:** Baseline characteristics of the sample of randomized controlled trial 2.

Baseline characteristics	Total sample (N=63)	CG^a^ (n=21)	IG^b^–PA^c^ (n=28)	IG–SB^d^ (n=14)	*F* or χ^2^ (df)	*P* value
Age (years), mean (SD)	58.68 (7.76)	57.67 (7.18)	59.00 (7.98)	59.57 (8.55)	0.29^e^ (2,60)	.75
Males, n (%)	16 (25)	6 (29)	4 (14)	6 (43)	4.19^f^ (2)	.12
University/college, n (%)	38 (60.32)	13 (62)	14 (50)	11 (79)	3.22^f^ (2)	.20
Body mass index (kg/m²), mean (SD)	25.91 (3.86)	25.29 (4.07)	26.14 (3.94)	26.34 (3.55)	0.40^e^ (2,58)	.68
Waist circumference (cm), mean (SD)	89.08 (12.89)	89.03 (14.69)	89.26 (11.22)	91.51 (13.93)	0.48^e^ (2,58)	.62

^a^CG: control group.

^b^IG: intervention group.

^c^PA: physical activity.

^d^SB: sedentary behavior.

^e^*F* value.

^f^χ^2^ value.

**Table 6:** Means and standard deviations for each of the behavioral outcomes in the three groups in randomized controlled trial 2.

Behavioral outcomes		CG^a^, mean (SD)		IG^b^–PA^c^, mean (SD)		IG–SB^d^, mean (SD)	
		Pre	Post	Pre	Post	Pre	Post
**LASA^e^ questionnaire**							
	Total sitting time (min/day)	414.21 (187.08)	421.52 (189.29)	378.39 (182.25)	335.25 (167.12)	615.00 (195.46)	549.69 (175.37)
**IPAQ^f^**							
	Total transport-related PA (min/day)	13.30 (15.29)	11.29 (12.93)	16.17 (35.33)	17.8 (13.32)	28.98 (40.10)	12.40 (15.48)
	Total household-related PA (min/day)	38.01 (41.71)	58.88 (70.27)	35.08 (36.66)	75.00 (78.41)	42.40 (51.65)	50.56 (37.63)
	Total leisure-related PA (min/day)	45.99 (68.62)	39.08 (39.40)	19.24 (27.66)	28.04 (31.95)	26.58 (31.16)	36.33 (37.84)
	Total PA (min/day)	134.57 (96.05)	117.55 (86.04)	109.39 (107.99)	168.93 (99.52)	98.42 (94.30)	104.18 (48.76)
	Moderate-to-vigorous physical activity (min/day)	65.20 (73.66)	64.25 (75.19)	63.88 (81.80)	106.39 (78.42)	36.12 (39.59)	60.61 (38.70)
**Accelerometer**							
	Number of breaks per day	13.61 (3.60)	12.81 (3.27)	13.01 (2.19)	12.02 (2.43)	15.02 (2.22)	14.10 (2.76)
	Length of sedentary bouts (min/day)	20.78 (2.50)	20.60 (3.02)	20.77 (2.08)	20.42 (2.50)	22.19 (2.19)	21.95 (2.66)
	Sedentary time (min/day)	482.41 (76.11)	460.11 (75.94)	472.52 (65.42)	450.09 (64.09)	512.94 (50.07)	486.94 (73.86)
	Light physical activity (min/day)	306.08 (89.43)	316.74 (74.81)	337.39 (80.84)	348.74 (81.03)	262.25 (53.62)	270.39 (61.93)
	Moderate physical activity (min/day)	29.13 (21.70)	22.70 (14.56)	24.35 (11.37)	17.14 (10.40)	26.26 (21.29)	28.27 (17.09)
	Moderate-to-vigorous physical activity (min/day)	29.33 (22.07)	23.51 (14.75)	24.96 (12.26)	17.23 (10.53)	28.96 (23.40)	30.94 (17.62)
	Total PA (min/day)	335.41 (91.72)	340.25 (73.81)	362.36 (82.76)	365.98 (87.64)	291.20 (66.53)	301.33 (73.21)
	Daily steps	7929.68 (2976.07)	7779.78 (2147.86)	8271.67 (2464.25)	7663.54 (2797.72)	7809.16 (3231.18)	8479.68 (3343.09)

^a^CG: control group.

^b^IG: intervention group.

^c^PA: physical activity.

^d^SB: sedentary behavior.

^e^LASA: Longitudinal Aging Study Amsterdam.

^f^IPAQ: international physical activity questionnaire.

**Table 7:** Time-by-group interactions and effect sizes for each of the behavioral outcomes in randomized controlled trial 2.

Behavioral outcomes		Time×group PA^a^ (ref: pre×CG^b^), beta (SE)	ES^c^ (IG^d^−PA vs CG)	Time×group SB^e^ (ref: pre×CG), beta (SE)	ES (IG−SB vs CG)
**LASA^f^ questionnaire**					
	Total sitting time (min/day)^g^	−0.06 (0.07)	−0.27	−0.14 (0.08)^h^	−0.37
**International physical activity questionnaire**					
	Total transport-related PA (min/day)^g^	0.26 (0.77)	0.13	−0.69 (0.88)	−0.43
	Total household-related PA (min/day)^g^	0.32 (0.65)	0.49	−0.26 (0.75)	−0.26
	Total leisure-related PA (min/day)^g^	0.54 (0.76)	0.32	0.48 (0.87)	0.35
	Total PA (min/day)^i^	73.85 (25.80)^j^	0.74	22.79 (28.92)	0.24
	Moderate-to-vigorous physical activity (min/day)^g^	0.52 (0.66)	0.55	0.53 (0.76)	0.48
**Accelerometer**					
	Number of breaks per day^i^	−0.30 (0.63)	−0.07	−0.36 (0.71)	−0.04
	Length of sedentary bouts (min/day)^i^	−0.12 (0.59)	−0.08	−0.11 (0.66)	−0.03
	Sedentary time (min/day)^i^	−4.76 (16.97)	−0.002	−8.90 (19.08)	−0.06
	Light physical activity (min/day)^i^	2.12 (12.79)	0.008	0.70 (14.29)	−0.04
	Moderate physical activity (min/day)^k^	2.36 (2.76)	−0.05	7.85 (4.17)^h^	0.40
	Moderate-to-vigorous physical activity (min/day)^k^	1.64 (2.78)	−0.11	7.50 (4.21)^h^	0.34
	Total PA (min/day)^i^	1.71 (14.43)	−0.02	7.23 (16.14)	0.07
	Daily steps^i^	−91.16 (553.35)	−0.17	763.22 (619.53)	0.26

^a^PA: physical activity.

^b^CG: control group.

^c^ES: effect size.

^d^IG: intervention group.

^e^SB: sedentary group.

^f^LASA:Longitudinal Aging Study Amsterdam.

^g^Gamma (log).

^h^*P*<.10.

^i^Gaussian (identity).

^j^*P*<.01.

^k^Gamma (identity).

**Table 8:** Time-by-group interactions and effect sizes for the personal determinants in randomized controlled trial 2.

Personal determinants	CG^a^, mean (SD)		IG^b^, mean (SD)		Time×group (ref: pre×CG), beta (SE)	ES^c^
	Pre	Post	Pre	Post		
Self-efficacy^d^	5.84 (2.67)	5.66 (2.08)	6.13 (1.82)	6.71 (1.71)	0.76 (0.39)^e^	0.37
Outcome expectancies^d^	7.27 (1.32)	7.32 (1.59)	7.31 (1.41)	7.30 (1.21)	0.30 (0.27)	−0.04
Risk perception^f^	2.64 (1.82)	2.57 (1.84)	3.79 (2.09)	3.83 (2.30)	−0.07 (0.08)	0.05
Action planning^d^	5.49 (2.27)	5.40 (2.29)	5.63 (2.24)	5.25 (2.16)	0.05 (0.65)	−0.13
Coping planning^d^	3.84 (2.69)	3.32 (1.78)	3.32 (2.30)	5.65 (2.04)	2.59 (0.50)^g^	1.19
Intention^d^	7.19 (2.11)	6.81 (2.57)	7.83 (1.88)	7.78 (1.70)	0.93 (0.51)^e^	0.17
Monitoring^d^	2.46 (2.40)	2.16 (1.69)	2.65 (1.77)	3.60 (2.36)	0.57 (0.34)^e^	0.65

^a^CG: control group.

^b^IG: intervention group.

^c^ES: effect size.

^d^Gamma (identity).

^e^*P*<.10.

^f^Gamma (log).

^g^*P*<.001.

## Discussion

### Efficacy of MyPlan 2.0

This study investigated the effect of a self-regulation–based eHealth and mHealth intervention (*MyPlan 2.0*) targeting an active lifestyle in two samples: adults having T2DM and adults aged ≥50 years. The study comprised two RCTs with an identical design. Although the pattern of results was overall in line with our hypotheses, the analyses revealed that the intervention only altered some of the outcomes. Indeed, this effect might be because of a lack of statistical power caused by the small samples in both the trials. The RCTs described here should, therefore, be considered pilot RCTs providing preliminary information regarding the potential effect of a HAPA-based eHealth and mHealth intervention in adults with T2DM and in adults aged ≥50 years.

The HAPA describes a number of personal determinants influencing the behavior change process. *MyPlan 2.0* affected various of these determinants. In RCT 1, an intervention effect in favor of the intervention group was found for action planning (borderline) and self-monitoring, but significant intervention effects favoring the control group were detected for risk perceptions and self-efficacy. In the RCT 2, intervention effects favoring the intervention group were detected for self-efficacy (borderline), intention (borderline), coping planning, and self-monitoring (borderline).

Some of these findings require additional attention. First, although targeted in the intervention, no intervention effect was found for outcome expectancies. This finding might be explained by a ceiling effect caused by the high levels of positive outcome expectancies at baseline in both RCTs. Indeed, our qualitative studies indicated that the users often have an extensive knowledge of the benefits of adopting an active way of living [29,30]. Second, although *MyPlan 2.0* does not provide the users with a pedometer or wearable automatically tracking the users’ behavior change, both RCTs identified intervention effects favoring the intervention group for monitoring. Avery et al found a negative effect of pedometer use on PA in people with T2DM and older adults, indicating that without additional support, these populations found it difficult to effectively reflect on the information provided by this self-monitoring tool [54]. Our results indicate that prompting the users to monitor their change and reviewing this change in the following session might be a feasible alternative to target self-monitoring in these samples. Third, the lack of effect for action planning in the RCT with adults aged ≥50 years was unexpected, as this determinant was targeted in each session. Sniehotta et al argued that action planning might play an important role for individuals who just started to put their intentions into actions, whereas coping planning would support individuals who moved further in the behavior change process to maintain their change under challenging conditions [55]. As the baseline levels of PA and SB of the RCT with the sample aged ≥50 years are quite close to the health norms [5], it is possible that this group already knew how to plan their actions and consequently, did not benefit from the action planning component. Similarly, considering the low levels of PA and high levels of SB at baseline in the RCT with adults with T2DM, the lack of evidence for coping planning could be explained by the fact that this group was not yet ready to optimally benefit from the coping planning component.

*MyPlan 2.0* focused on altering the users’ level of PA or SB. In RCT 1, borderline significant intervention effects favoring the PA intervention group were found for self-reported daily sitting and accelerometer-assessed MPA and MVPA. This is an important result as a previous study by Silfee et al, testing a self-regulation–based intervention targeting PA in adults with T2DM, did not show behavioral effects despite the positive effect on personal determinants for change (including self-monitoring) [56]. In RCT 2, an intervention effect favoring the PA intervention group was found for self-reported total PA. This effect is in line with the previous research with *MyPlan 1.0* in recently retired older adults [57]. The lack of evidence for intervention effects on self-reported domain-specific PA in both RCTs is in line with our hypotheses and can be explained by the fact that *MyPlan 2.0* allows the users to select each session a different PA-domain that is at that moment most relevant to them rather than imposing a specific domain.

In RCT 1, an intervention effect favoring the SB intervention group was found for accelerometer-assessed daily breaks from sedentary time. To our knowledge, *MyPlan 2.0* is the first eHealth and mHealth intervention targeting sedentary behavior in adults with type 2 diabetes. Considering the health effects of breaking up periods of prolonged sitting in adults with T2DM [58], this result warrants further research regarding eHealth interventions targeting sedentary behavior in adults with type 2 diabetes. In RCT 2, an intervention effect favoring the SB intervention group was detected for self-reported daily sitting time (borderline). This finding is in line with the research by Stephenson et al, indicating that technology enhanced interventions are able to reduce sedentary behavior [59]. Although it is assumed that sedentary behavior will be replaced by LPA rather than MVPA [60], intervention effects favoring the SB intervention group were found for MPA (borderline) and MVPA. Similarly, Gardiner et al found that their intervention to reduce and break up sedentary time in older adults resulted in changes in sedentary time, breaks from sedentary time, LPA, and MVPA [61].

Overall, the lack of intervention effects reaching statistical significance could be interpreted as disappointing. However, one has to keep in mind the following issues that may have led to an underestimation of our effects. First, in keeping with the self-regulation literature, *MyPlan 2.0* motivated the users to set and pursue their own goals. Consequently, the set goals could differ strongly between as well as within the participants (ie, each session the participants could select a different goal) on 4 aspects: chosen behavior (eg, MVPA vs LPA), ambitiousness (eg, reaching 500 vs 5000 additional steps), setting (eg, leisure time vs transport), and time frame (eg, every day of the week vs in the weekend). This might have lowered the chance of finding an effect. However, this approach was believed to be better and more sustainable. It would lead to more success experiences and a greater willingness to continue with the process of behavioral change. From a methodological point of view, we may, therefore, recommend targeting one type of behavior (eg, decreasing sitting time) that can be performed in a wide variety of settings. This approach will allow (1) the users to create personal goals (ie, create a sense of goal ownership) and (2) the researchers to select the most appropriate measurement methods to detect alterations in the targeted behavior. Second, as accelerometers are not able to capture posture, these devices tend to have problems to distinguish between sedentary time and light-intensity PA [62]. This could imply that some of the accelerometer-assessed breaks do not automatically reflect posture change from sitting to standing. Furthermore, previous research already indicated that the agreement between self-reported and objective measurements of PA is limited [63]. Indeed, instead of creating a hierarchy of preferred measures, objective and self-report measures should be considered distinct rather than interchangeable [64]. Finally, our limited power caused by the small samples might have hindered a number of effects to reach statistical significance.

### Attrition Levels in MyPlan 2.0

Web-based interventions are characterized by high levels of attrition [65]. More than 70% of *MyPlan 1.0* users did not complete the intervention [66,67]. In RCT 1, 36% (13/36) of the participants receiving *MyPlan 2.0* did not complete the intervention. In RCT 2, this was 19% (8/42). These massive reductions in attrition might be explained by the iterative adaptations that were made to the program to increase engagement and by the fact that the participants were phoned on a weekly basis. However, in both RCTs, we found that the participants receiving the intervention were still more likely to quit compared with those in the control group. Furthermore, in RCT 2, we found that dropout was higher in users with a lower level of education. These findings were disappointing as we, being aware of this issue, purposefully conducted a series of studies to adapt the intervention’s content to this target population [29,30,32]. Yardley et al argue to make a distinction between the micro (engagement with intervention itself) and macro (engagement with the behavior change process to reach the set goals) level of engagement to create effective engagement (ie, *sufficient engagement with the intervention to reach the desired outcomes* [68]) rather than simply more engagement. This idea is in line with the hypothesis of Eysenbach stating that the users need to experience the added value of using the Web-based intervention to prevent attrition [65]. Consequently, not only investigating whether the users like the program itself but also identifying how they put the learned techniques into practice and which variables (eg, level of education) moderate this process might be a fruitful avenue to (1) decrease the level of attrition and (2) increase the effectiveness of Web-based interventions in the future.

### Strengths and Limitations of the Study

This study has several strengths. First, several studies have assessed the effect of internet-based interventions on SB in the general population [12,59]. To our knowledge, this is the first study testing a Web-based intervention targeting SB in adults with T2DM. Second, by also assessing the HAPA-based determinants for change, we were able to check whether the implemented behavior change techniques effectively altered the users’ personal determinants for change. Finally, by using self-report as well as objective measurements, a more nuanced view of the effects was presented. However, it should be acknowledged that the self-report and objective measures did not represent the same time frame.

There are also a number of limitations. First, no power analysis was conducted for RCT 2. Second, the small sample sizes made it difficult to detect statistically significant effects. Third, a waiting-list rather than a placebo control group was created. Consequently, we are not certain whether the detected intervention effects were actually caused by the active ingredients of the intervention. Furthermore, informing a participant that he or she is allocated to a waiting-list control group might have influenced his/her behavior (eg, participants of the control group might have felt reluctant to alter their behavior as they knew they would receive support later). Indeed, previous research has shown that trials using a waiting-list control condition might overestimate treatment effects [69]. Fourth, to analyze the effect of *MyPlan 2.0* on the HAPA-based personal determinants, no distinction was made between the two intervention groups (ie, they were combined into one group). Consequently, it was not possible to investigate whether the intervention effects for the personal determinants altered according to the chosen behavior. Fifth, the users were contacted each week to assess potential negative effects (eg, hypoglycemia). Furthermore, the users who forgot to log in for the following session were contacted by the researcher to inform them about the awaiting session. These phone calls might have motivated the participants to stay in the study and to complete the intervention. Consequently, the detected attrition rates might be an underestimation of the actual attrition rates of the program. Finally, the effects reported here reflect short-term changes. However, a third wave of data collection at 10 months post baseline will be performed.

### Conclusions

To conclude, this study suggests that a self-regulation–based Web-based intervention has the potential to alter levels of PA and SB in adults with T2DM and in adults aged ≥50 years. However, further research with larger samples is needed to confirm the consistency of these findings.

## Appendix

Multimedia Appendix 1 Overview of adaptations made to “MyPlan 2.0” based on the user-based studies.

Multimedia Appendix 2 Screenshots of “MyPlan 2.0”.

Multimedia Appendix 3 CONSORT-EHEALTH checklist (V 1.6.1).

## Abbreviations

BIC: Bayesian information criterion
BMI: body mass index
CG: control group
CPM: counts per minute
ES: effect size
IG: intervention group
IPAQ: international physical activity questionnaire
LASA: Longitudinal Aging Study Amsterdam
LPA: light physical activity
MPA: moderate physical activity
MVPA: moderate-to-vigorous physical activity
PA: physical activity
RCT: randomized controlled trial
SB: sedentary behavior
T2DM: type 2 diabetes mellitus
VPA: vigorous-intensity physical activity
