# Supplementary material for: Efficacy of a Self-Regulation–Based Electronic and Mobile Health Intervention Targeting an Active Lifestyle in Adults Having Type 2 Diabetes and in Adults Aged 50 Years or Older: Two Randomized Controlled Trials
Source: J Med Internet Res. 2019 Aug 2;21(8):e13363. doi: 10.2196/13363 (PMC6696857; doi:10.2196/13363)
Supplement: Multimedia Appendix 1 [file jmir_v21i8e13363_app1.pdf]

Overview of the adaptations in ‘MyPlan 2.0’ based on the results of the user-based studies.

<table>
<tr><th>Studies</th><th>Lessons Learned</th><th>Implementation of the findings in ‘MyPlan 2.0’</th></tr>
<tr><td colspan="3">Studies performed with ‘MyPlan 1.0’</td></tr>
<tr><td>Attrition Study

DOI: https://doi.org/10.2196/jmir.7277</td><td>High levels of attrition already occur at the first session of ‘MyPlan 1.0’.

Potential users of ‘MyPlan 1.0’ are reluctant to create an account.</td><td rowspan="2">The sign-up page of ‘MyPlan 2.0’ indicates that users’ email address will only be used to send the four follow-up emails.

Besides adding diabetes-specific information regarding the importance of checking blood glucose levels when being physically active no components were added or removed for the version targeting adults with type 2 diabetes.

In order to offer a more personalised approach, success stories based on the user’s age and gender were added to the intervention.

Rationales for each proposed behaviour change technique were added. For example, the website explains why coping planning is important and how it can help users in the behaviour change process.

Research-related questions (e.g. questions assessing participants’ personal determinants for change) were removed and lengthy text pages providing information on the benefits of adopting an active lifestyle were replaced by quizzes.

Instead of asking participants to send their plan to friends or colleagues optional pages providing information on how social support can be obtained from friends, family or colleagues were created.

Participants are no longer required to create implementation intentions. The plan is now made more specific by asking users</td></tr>
<tr><td>Think Aloud Study

DOI: https://doi.org/10.1371/journal.pone.0190020</td><td>The remarks of the sample with type 2 diabetes were similar to those of the sample from the general population.

Participants stated that ‘MyPlan 1.0’ was poorly tailored to their situation.

Participants did not understand the usefulness of several behaviour change techniques implemented in ‘MyPlan 1.0’.

Participants stated that ‘MyPlan 1.0’ was not time-efficient and described the programme as a long questionnaire rather than an intervention.

Participants were reluctant to send their plans to friends and family.

Participants experienced difficulties to create implementation intentions (i.e. ‘if-then plans’).

Participants stated that they did not like the lay-out of the website.

Participants stated that a mobile application would be useful in their behaviour change process.</td></tr>
</table>

		questions such as when, where and how many times they will perform the selected behaviour (e.g. being physically active during leisure time).<br><br>Questions assessing participants' level of physical activity/sedentary behaviour were not removed as these were needed to provide tailored feedback. However, the number of these questions was restricted to a minimum.<br><br>To create a more enjoyable interface, lengthy text pages were deleted and more images were added.<br><br>A mobile application providing daily support was created.
**Studies performed with 'MyPlan 2.0'**		
Interview Study (users with type 2 diabetes)<br><br>DOI: https://doi.org/10.3390/ijerph15050954	Participants appreciate the time-efficiency and user-friendliness of 'MyPlan 2.0'.<br><br>Participants did not learn new information by going through the quiz, but became more aware of the importance of increasing physical activity/decreasing sedentary behaviour.<br><br>Participants experience the website as personally relevant.<br><br>Participants felt motivated by the action planning component as they knew they would receive feedback in the next session.<br><br>Participants experienced problems to complete the coping planning component.<br><br>Participants liked to go through the programme together with their partner.	Users no longer need to indicate whether they selected physical activity or sedentary behaviour at each of the follow-up sessions. They are immediately guided to the correct version.<br><br>A button saying "need inspiration?" was added to open-ended questions of the coping planning component. After clicking on this button participants are guided to a page with an extensive list of potential barriers and feasible solutions to overcome these barriers.<br><br>A printable weekly overview was added to the component prompting users to self-monitor their behaviour change. Furthermore, the mobile application offers a specific component to help users monitor their changes.<br><br>The mobile application allows people to alter the goals created on the website.

Mixed Methods Study (users from the general population)<br><br>DOI: https://doi.org/10.2196/10412	Participants indicated that the website is user-friendly and time-efficient.<br><br>The difference between physical activity and sedentary behaviour was unclear to many users<br><br>Users would like to see new content per session and more interaction.<br><br>Almost all users experienced the website as personally relevant.<br><br>The implementation of the action planning component was feasible: users liked the specific questions, few users stated unachievable plans and many stated that they were able to reach their goals, at least partially. However, some users found it difficult to create plans a week in advance.<br><br>Users experienced difficulties to complete the coping planning component and many coping plans were of poor quality.<br><br>Although prompted to self-monitor their changes, many users did not keep track of their behaviour change.	To achieve a higher level of interaction, an additional page with recommendations was created for people who indicated that they were not able to reach their goal.
