# Supplementary material for: Efficacy of a Self-Regulation–Based Electronic and Mobile Health Intervention Targeting an Active Lifestyle in Adults Having Type 2 Diabetes and in Adults Aged 50 Years or Older: Two Randomized Controlled Trials
Source: J Med Internet Res. 2019 Aug 2;21(8):e13363. doi: 10.2196/13363 (PMC6696857; doi:10.2196/13363)
Supplement: Multimedia Appendix 2 [file jmir_v21i8e13363_app2.pdf]

Screenshots of ‘MyPlan 2.0’

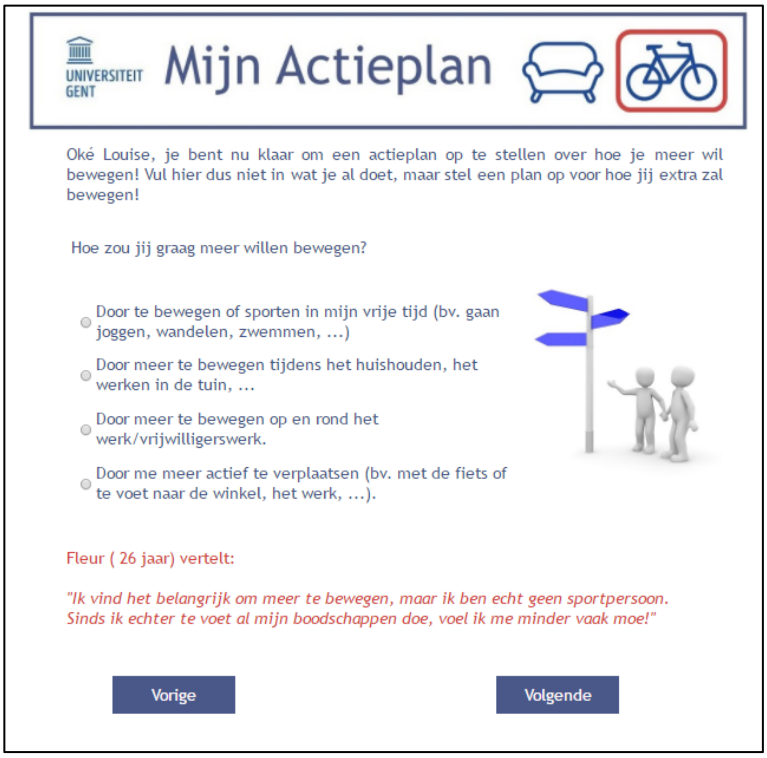

Figure 1. Action Planning (website)

Users are prompted to make their plan more specific by indicating what kind of physical activity they aim to do in the following week. The text in red is a success story : “Fleur (26 years old) tells: I consider it important to be more physically active, but I am not a sporty person. Since I do all my groceries by foot, I feel less tired during the day!”.

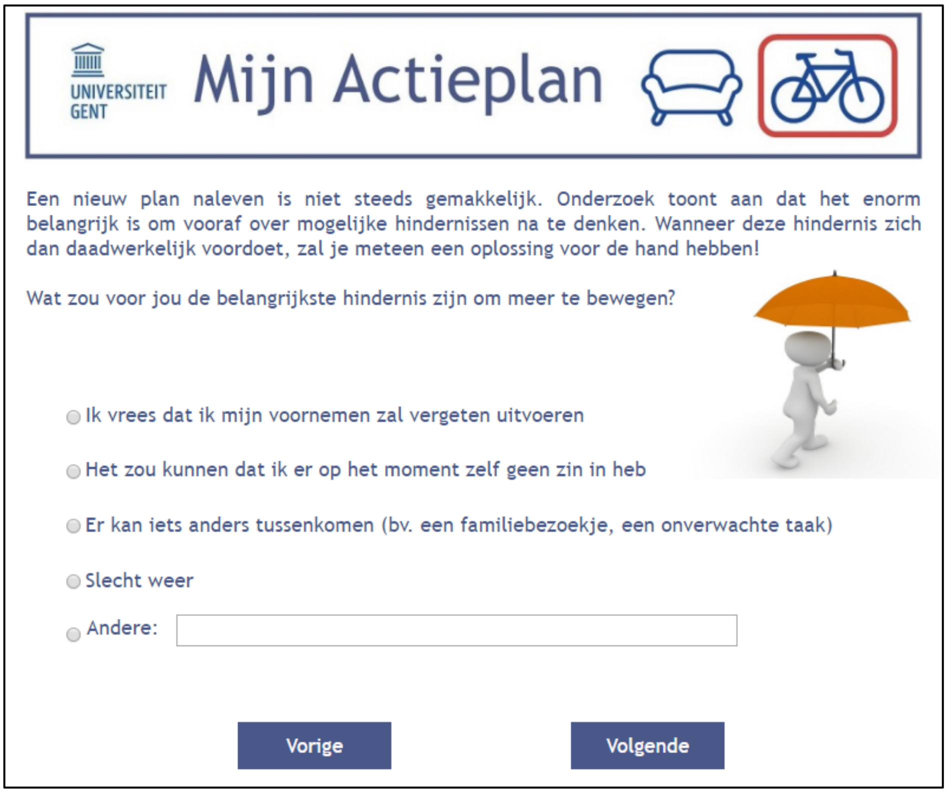

Figure 2. Coping planning (website)

Translation: “Living up to a new plan is not always easy. Research shows that it is really important to consider potential barriers in advance. When this barrier appears, you will have an immediate solution at hand!” Users are asked to select barriers they might face in the upcoming week. Afterwards, solutions based on the selected barriers are shown and users need to indicate which solution they will apply.

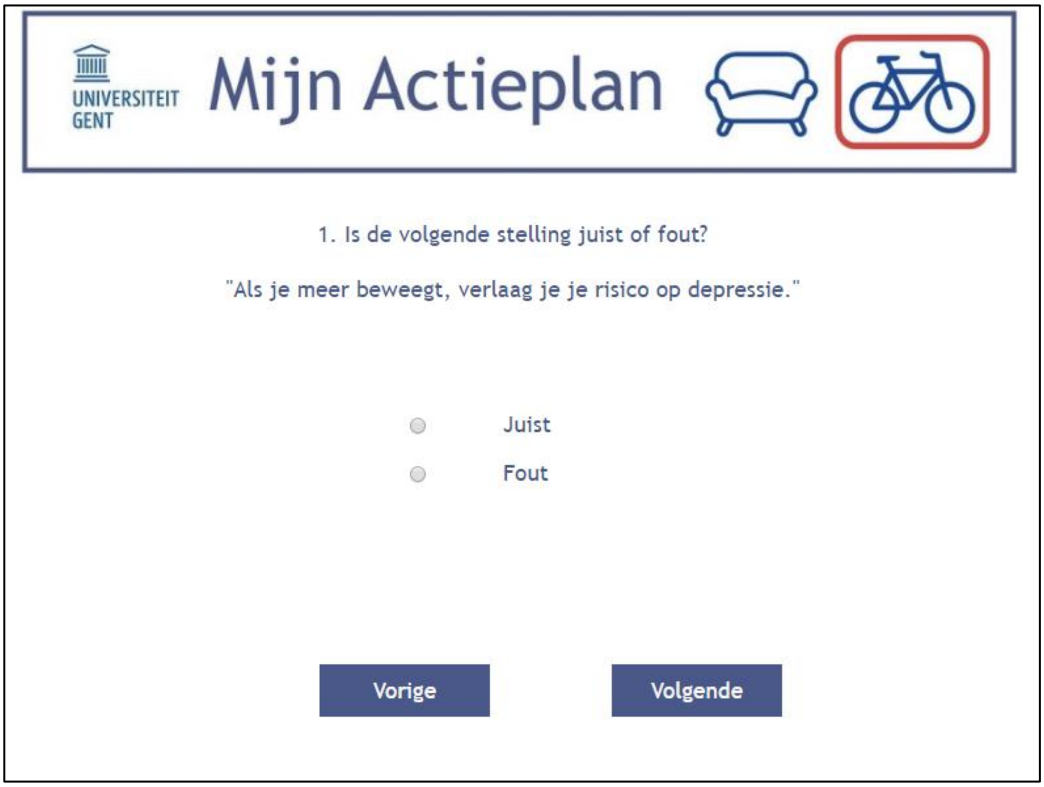

Figure 3. Example of a quiz question (website)

Translation: "Is the following statement true or false? I you are more physically active, you have a lower chance on developing a depression."

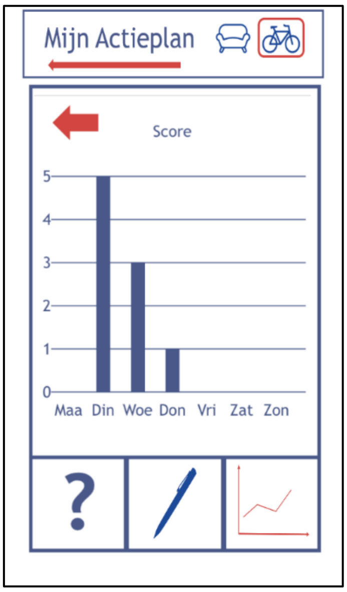

Figure 4. Monitoring health behaviours (mobile application)

Users can indicate for each day to which extent they were able to meet their goal (0 = not at all and 5 = completely).

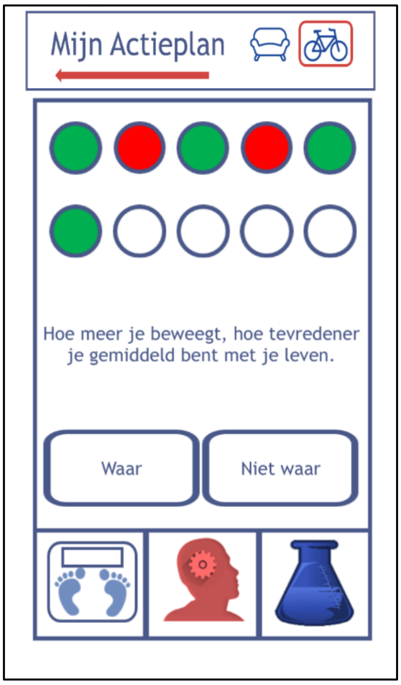

Figure 5. Quizzes (mobile application)

Translation: “In general, the more people are physically active, the more happy they are with their lives.” Blue boxes: “True” and “False”.

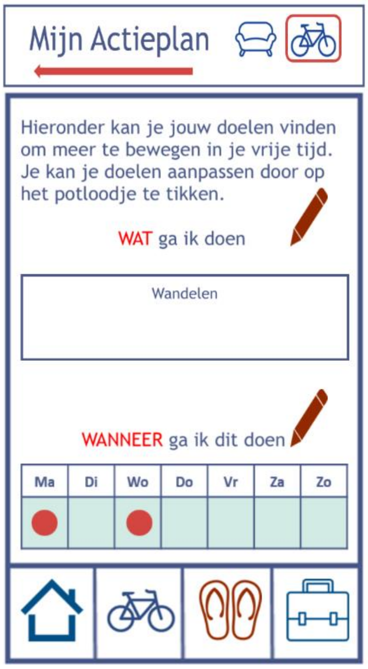

Figure 6. Revising and adapting plans (mobile application)

Translation: “Below you can find your goals to be more physically active during leisure time. You can adapt your goals by tapping on the pencil icon.”

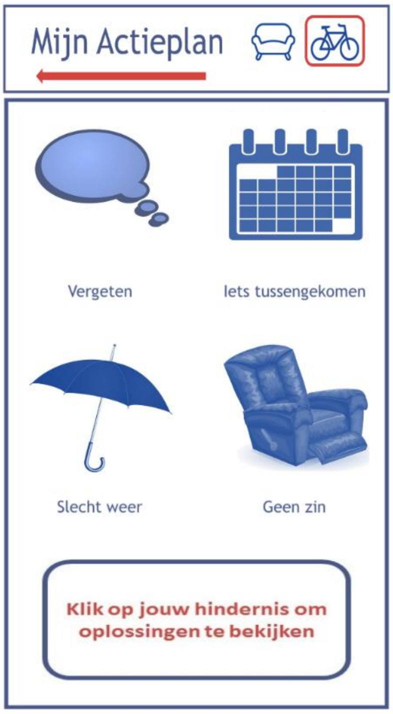

Figure 7. Coping planning (mobile application)

Translation: “Tap on a barrier to see potential solutions”.
